# Books and Magazines

**Published:** 1910-08

**Authors:** 


### Books and Magazines.

THE DIAGNOSIS OF SMALLPOX. By T. F. Ricketts, M. D., M. R. C. P., D. P. H., Medical Superintendent of the Smallpox Hospitals and of the River Ambulance Service of the Metropolitan Asylums Board. Illustrated from photographs by J. B. Byles, M. B., B. C., F. R. C. S., D. P. H. Many charts, colored photographs taken from life, and black-and-white illustrations. Octavo. Prica, $6.00, net; postpaid, $6.25. Funk & Wagnalls Co., New York. 1910.

"Every possible phase and distribution of the variolous eruptions are shown in a series of photographic plates which are so extraordinarily good in themselves, and so admirably reproduced, that they are to a great extent capable of affording information which, as a rule, can only be acquired at the bedside of an enormous number of patients."—*Edinburgh Medical Journal.*

---

SERUMS, VACCINES, AND TOXINES, IN TREATMENT AND DIAGNOSIS.—(Mcdern Methods of Treatment Series.) By William Cecil Bosanquet, M. A., M. D., F. R. C. P., Assistant Physician to Charing Cross Hospital, and John W. Eyre, M. D., Bacteriologist to Guy's Hospital and in charge of Vaccine Department. Octavo, cloth, 344 pp. Price, $2.00, net; postpaid, $2.10. Funk & Wagnalls Co., New York. 1910.

Contents: "Immunity and Resistance to Disease," "Preparation and Administration of Serums and Vaccines," "Serums and Toxines in the Diagnosis of Disease," "Diphtheria," "Tetanus," "Snake-Bite," "Smallpox and Vaccinia," "Hydrophobia (Rabies)," "Plague," "Enteric Fever," "Cholera," "Affections Due to Streptococci," "Tuberculosis," "Other Conditions Treated by Antibacterial Methods," "Anthrax and Glanders," "Malignant Tumors." Appendix I—"The Therapeutic Use of Normal Serum (Horse Serum, etc.)." Appendix II—"List of Preparations." Index.

Hygiene and Public Health. By H. A. Whitelegge, C. B., M. D., and George Newman, M. D. New edition revised and rewritten. A concise summary of the current knowledge of public health in every branch of the public service; an elementary text-book for the student. Thirteenth thousand. 16mo., cloth, 636 pp. Illustrated. Price, $1.75, net; postpaid, $1.85. Funk & Wagnalls Co., New York. 1910.

"The carefulness with which the revision of this edition has been carried out is apparent on every page."—*British Medical Journal.*

---

Radiumtherapy. By Dr. Louis Wickham and Dr. Degrais. Translated from the French by S. Ernest Dore, M. D., with an introduction by Sir Malcolm Morris, K. C. V. O., one of the physicians to King Edward VII. Octavo, cloth. 300 pp. $5.00, net; postpaid, $5.15. Funk & Wagnalls Co., New York. 1910.

This is the latest and the most complete work on this subject. The authors are well known in France as among the first to use this new remedial agent and they have patiently studied and experimented for years; this book is the result of their research and practice and may be said to contain the last word on the subject up to the present. Sir Malcolm Morris, who is himself one of the foremost skin specialists in the world, says in his introdution to this book that Dr. Wickham "is the true pioneer in the new region now gradually being opened up of the therapeutic application of radium," and that "nothing can deprive Dr. Wickham of the glory of having laid the foundation stone of scientific radium-therapy." The present translation contains "a large number of new facts illustrating operative methods and therapeutic results" which are not in the French edition, although the latter appeared only a year ago.

---

SYPHILIS. By Sir Jonathan Hutchinson, F. R. S., LL. D., F. R. C. S. Consulting Surgeon to the London Hospital, and to the Royal London Ophthalmic Hospital. Tenth thousand Entirely new edition and largely rewritten in 12 colored and 24 black-and-white plates. Octavo, cloth. 600 pp. Price, $3.00, net; postpaid, $3.17. Funk & Wagnalls Co., New York. 1910.

This volume has long been out of print, the publication of the present edition having been delayed by the author's desire to take full advantage of the demonstration of the *Spirochæta pallida* as the immediate cause of syphilis, with the experimental work which has followed that discovery. He suggests that the spirochete is not to be regarded as representing the sum total of syphilis, and that the complex group of symptoms which results from a spirillum invasium is often more importantly due to the patient's proclivities than to the parasite itself. Among other questions dealt with in the volume is the use of the salts of arsenic in the treatment of syphilis.

